# *Leishmania donovani*: Immunostimulatory Cellular Responses of Membrane and Soluble Protein Fractions of Splenic Amastigotes in Cured Patient and Hamsters

**DOI:** 10.1371/journal.pone.0030746

**Published:** 2012-01-26

**Authors:** Shraddha Kumari, Pragya Misra, Rati Tandon, Mukesh Samant, Shyam Sundar, Anuradha Dube

**Affiliations:** 1 Parasitology Division, Central Drug Research Institute, CSIR, Lucknow, Uttar Pradesh, India; 2 Department of Immunology, Rikshospitalet-Radiumhospitalet Medical Centre, Institute for Cancer Research, Montebello, Oslo, Norway; 3 Department of Medical Biology, Research Center in Infectious Diseases, CHUL Research Center, Laval University, Quebec, Canada; 4 Department of Medicine, Institute of Medical Sciences, Banaras Hindu University, Varanasi, Uttar Pradesh, India; University of Oklahoma Health Sciences Center, United States of America

## Abstract

Visceral leishmaniasis (VL), caused by the intracellular parasite *Leishmania donovani*, *L. chagasi* and *L. infantum* is characterized by defective cell-mediated immunity (CMI) and is usually fatal if not treated properly. An estimated 350 million people worldwide are at risk of acquiring infection with *Leishmania* parasites with approximately 500,000 cases of VL being reported each year. In the absence of an efficient and cost-effective antileishmanial drug, development of an appropriate long-lasting vaccine against VL is the need of the day. In VL, the development of a CMI, capable of mounting Th1-type of immune responses, play an important role as it correlate with recovery from and resistance to disease. Resolution of infection results in lifelong immunity against the disease which indicates towards the feasibility of a vaccine against the disease. Most of the vaccination studies in Leishmaniasis have been focused on promastigote- an infective stage of parasite with less exploration of pathogenic amastigote form, due to the cumbersome process of its purified isolation. In the present study, we have isolated and purified splenic amastigotes of *L. donovani*, following the traditional protocol with slight modification. These were fractionated into five membranous and soluble subfractions each i.e MAF1-5 and SAF1-5 and were subjected for evaluation of their ability to induce cellular responses. Out of five sub-fractions from each of membrane and soluble, only four viz. MAF2, MAF3, SAF2 and SAF3 were observed to stimulate remarkable lymphoproliferative, IFN-γ, IL-12 responses and Nitric Oxide production, in *Leishmania*-infected cured/exposed patients and hamsters. Results suggest the presence of Th-1 type immunostimulatory molecules in these sub-fractions which may further be exploited for developing a successful subunit vaccine from the less explored pathogenic stage against VL.

## Introduction

Visceral leishmaniasis (VL) is the most fatal among the complex of leishmaniases and is caused by the invasion of the reticuloendothelial system by the hemoflagellate protozoan parasite, *Leishmania donovani*, *Leishmania infantum*, and *Leishmania chagasi*. VL is endemic in 62 countries, with a total of 200 million people at risk and an estimated 500 000 new cases arising each year worldwide [1]. Toxicity of currently available drugs alongwith limited efficacy and emerging resistance to these antileishmanials is an encumbrance for ongoing available treatment regimens and is of significant concern [2]. It is well known that during active VL there is profound immunosuppression and development of an effective CMI, capable of mounting Th1-type of immune response, plays an important role in controlling *Leishmania* infections in experimental and human VL [3], [4]. Thus, in the abovesaid scenario, development of an effective vaccine against leishmaniasis should be a priority of tropical disease research.

Further, in areas endemic for visceral leishmaniasis, there appears to be a significant population of individuals who have had in-apparent or self-resolving infection with *L. donovani* and are presumably resistant to visceral disease. This may provide a rationale for designing immunoprophylactic strategies against VL. Unfortunately, the development of vaccines has been hampered by significant antigenic diversity along with the fact that the parasites have a digenetic life cycle in at least two hosts- vector and human, there are differences in the host cell responses following interaction with either promastigote or amastigote forms or their antigens [5], [6]. Since the promastigote form can be readily cultivated in cell-free media at room temperature (23 to 27°C), the majority of studies have investigated the host cell response initiated by *Leishmania* promastigotes [7], [8]. Our earlier studies have also aimed at identifying promastigote antigens (membranous as well as soluble) from a field isolate of *Leishmania* spp. for the molecules having ability to stimulate Th1-type response, which is known to be the major defense mechanism against *Leishmania* infection [3], [4], [9], [10], [11]. It has been reported that some T-cell epitopes that are protective in the murine host do not elicit an immune response in human [12], emphasizing the importance of evaluating leishmanial antigens for their cellular immune responses in patients' samples. Keeping this view, we made an attempt to translate and compare the observations in hamster model, a perfect model mimicking progressive human VL, with patients' samples. Both the membranous as well as soluble antigens induced significant proliferative responses and IFN-γ production in lymphocytes isolated from cured VL patients and cured hamsters [13]. Encouraged with the results we further fractionated promastigote soluble lysate into 5 fractions and assessed these for their immunogenicity as well as immunoprophylactic efficacy. Out of these, fraction # 2 (F2) ranging from 68–97.4 kDa elicited considerably good T cell responses in individuals who had recovered from VL and endemic contacts as well as in cured hamsters [13], [14]. In addition, F2 fraction showed considerably good prophylactic activity together with BCG against *L. donovani* challenge [13]. Thus, generation of similar type of responses in both the hosts against leishmanial antigens encouraged the fact that findings obtained with hamster model can be translated clinically in future.

Abovesaid observations with promastigotes led to the necessity and importance to dissect the membrane and soluble proteins of amastigote form, which is actually responsible for disease pathogenesis, and thus can be a more potential yet unexplored reservoir of immunostimulatory antigens. The reason behind less exploration of splenic amastigotes for vaccine development is the time-consuming and low yielding isolation of this stage, either from lesions or by *in vitro* cultivation in macrophages and macrophage-like cell lines, alongwith the major issue of contamination of host components. However, the availability of axenic cultures of several *Leishmania* strains [15], [16], [17] has allowed detailed studies of biological and immunologic functions of amastigote proteins. But these axenic forms can be grown only for the few Leishmania species- particularly of cutaneous forms, and although they display a number of biochemical markers of the intracellular stage [18], they fail to synthesize some major products of true amastigotes, *e.g.* the secreted amastigote-specific proteophosphoglycans in the case of *Leishmania mexicana.* Moreover, recent genome-wide mRNA profiling suggests that axenic amastigotes are more closely related to promastigotes than to *ex-vivo* isolated amastigotes [19] emphasizing the need to shift the studies with ‘true’ amastigotes to get into its authentic data.

The present study was conducted with the same line of work, as was performed with promastigote, in splenic intracellular amastigote stage. Herein, we fractionated membrane as well as soluble fractions of amastigotes into five sub-fractions according to their molecular weights and evaluated these for their ability to stimulate cellular responses in the macrophages and lymphocytes of *Leishmania* infected and cured patients as well as hamsters to identify and ascertain the specific immunostimulatory fractions or constituents in them.

## Results

### *L. donovani* amastigotes isolated from infected spleen were free of host contaminants

**Figure 1:**
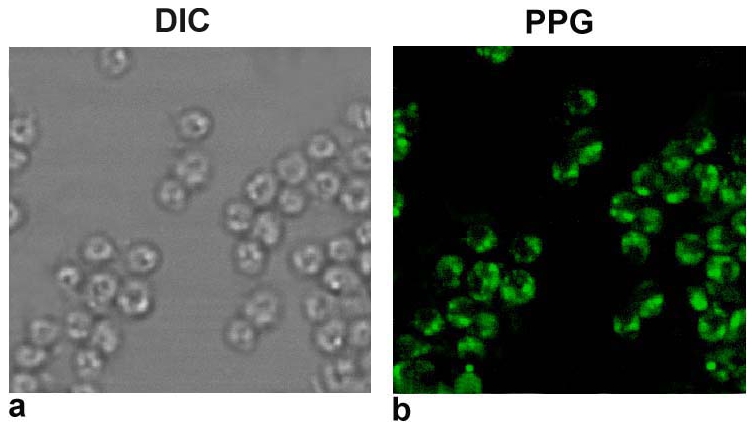
Immunolocalization of PPG in pure amastigotes derived from splenic tissue by confocal laser scanning microscopy. (a) DIC image (Differential interference contrast image) of purified amastigotes in confocal microscopy which is similar to that obtained by phase contrast microscopy but without the bright diffraction halo (b) Fluorescent image of purified amastigotes using anti-PPG antibody with secondary anti-rabbit FITC labeled IgG.

Since host contamination is the major issue with all the studies related to splenic amastigotes it was essential to ensure the removal of host debris from the amastigote suspension, isolated from infected spleen. This was done by confocal microscopy using antibody against proteophosphoglycan which we have used for the same purpose earlier also [29]. Amastigotes showed bright green fluorescence throughout their outer surface and flagellar pocket (Fig. 1.). The control antibodies (normal rabbit serum) used showed no fluorescence. As observed in DIC as well as fluorescent images, there was no host contaminants observed in amastigotes purified by this protocol.

### Isolation and SDS-PAGE analysis of sub-fractions

**Figure 2:**
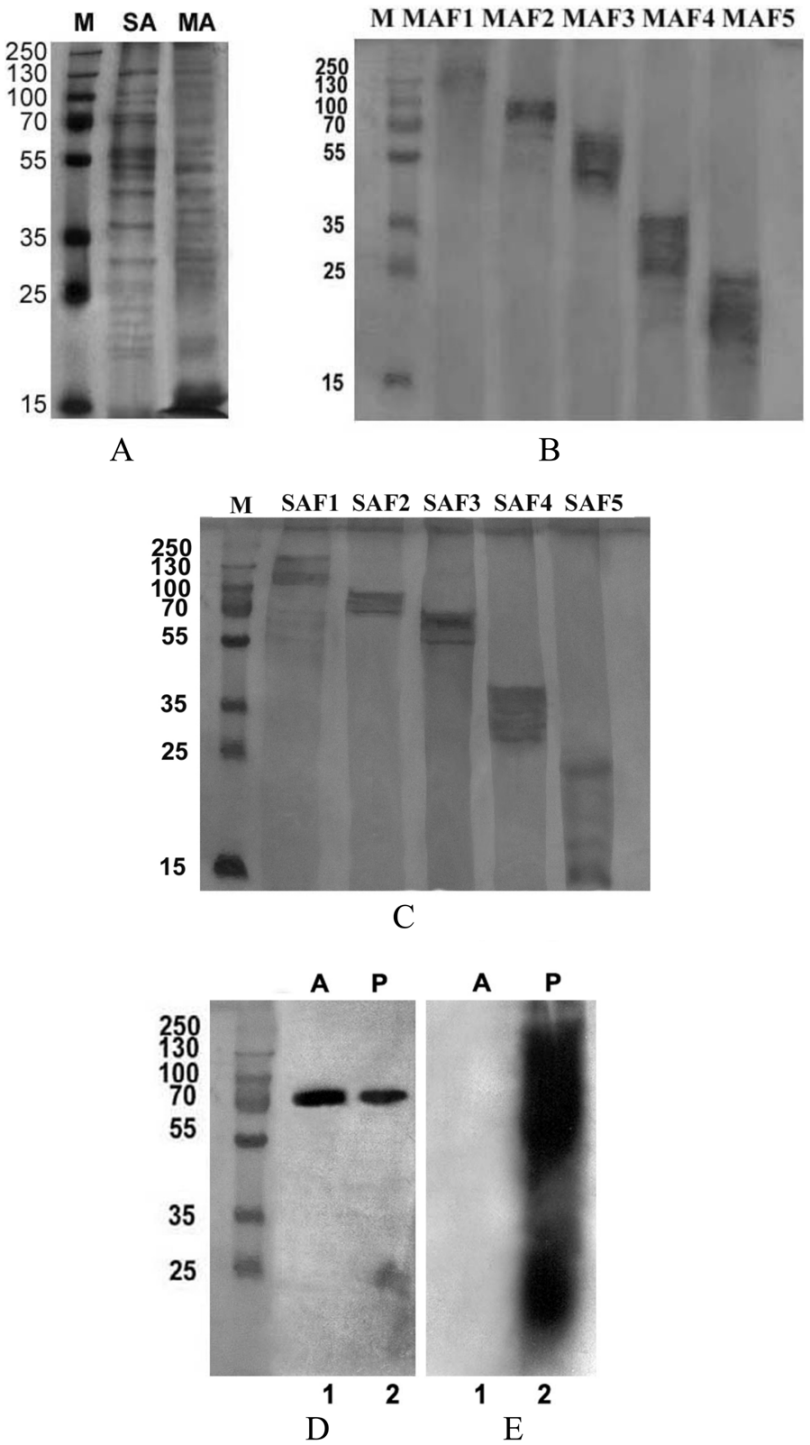
Silver stained 12% SDS-PAGE gels showing soluble antigenic fraction/SA & membrane antigenic fraction/MA (A) as well as purified sub-fractions of MA (B) and SA (C) according to their molecular weight. Western blot analysis of amastigotes and promastigotes using (i) GRP-78 antibody as loading control (D), (ii) mouse monoclonal LPG against promastigotes (E). Lane 1: Amastigote lysate; Lane 2: promastigote lysate.

The purified amastigote suspension was fractionated into MA and SA and further sub-fractionated into five sub-fractions to homogeneity by electro-elutor (Fig. 2 A). Total 5 sub-fractions were collected for MA and SA each (Fig. 2 B, C). The purity and accuracy of the samples was assessed by SDS-PAGE, followed by silver staining. Five discrete sub-fractions for MA and SA were MAF1, MAF2, MAF3, MAF4, MAF5 (MW ranges of 205-97, 97-68, 68-43, 43-29, 29-10 respectively) and SAF1, SAF2, SAF3, SAF4, SAF5 (MW ranges of 205-97, 97-68, 68-43, 43-29, 29-10 respectively).

### Verification of amastigotes proteins through expression of LPG and A2 by Western blot

**Figure 3:**
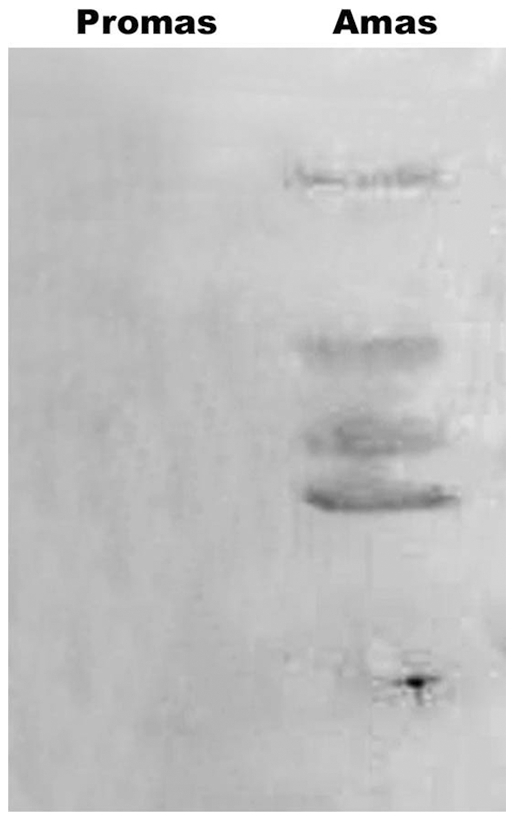
Western blots analysis of amastigotes and promastigotes using mouse monoclonal A2 antibody against amastigotes. Lane 1: promastigote lysate; Lane 2: Amastigote lysate.

Further, to ensure that the proteins we have used in the study are derived from amastigotes, we took promastigote as well as amastigote specific marker antibodies against LPG as well as A2 respectively. LPG of promastigotes is antigenically and biochemically different from amastigotes [30]. Similarly, A2 protein is a stage specific protein of amastigotes [31]. Immunoblot analysis of whole cell lysate of splenic amastigotes and promastigotes with monoclonal antibody against promastigotes LPG revealed the presence of LPG in promastigotes but its absence in amastigotes (Fig. 2E).The loading control indicated a similar abundance of the endoplasmic reticulum protein grp78 in both the stages of parasites(Fig. 2D). Similarly, immunoblot analysis using monoclonal antibody against amastigotes A2 revealed its presence in amastigotes only (Fig. 3).

### Evaluation of Th1 stimulatory cellular responses of potential fractions with PBMCs of cured/endemic *Leishmania* patients

#### Lymphoproliferative response

**Figure 4:**
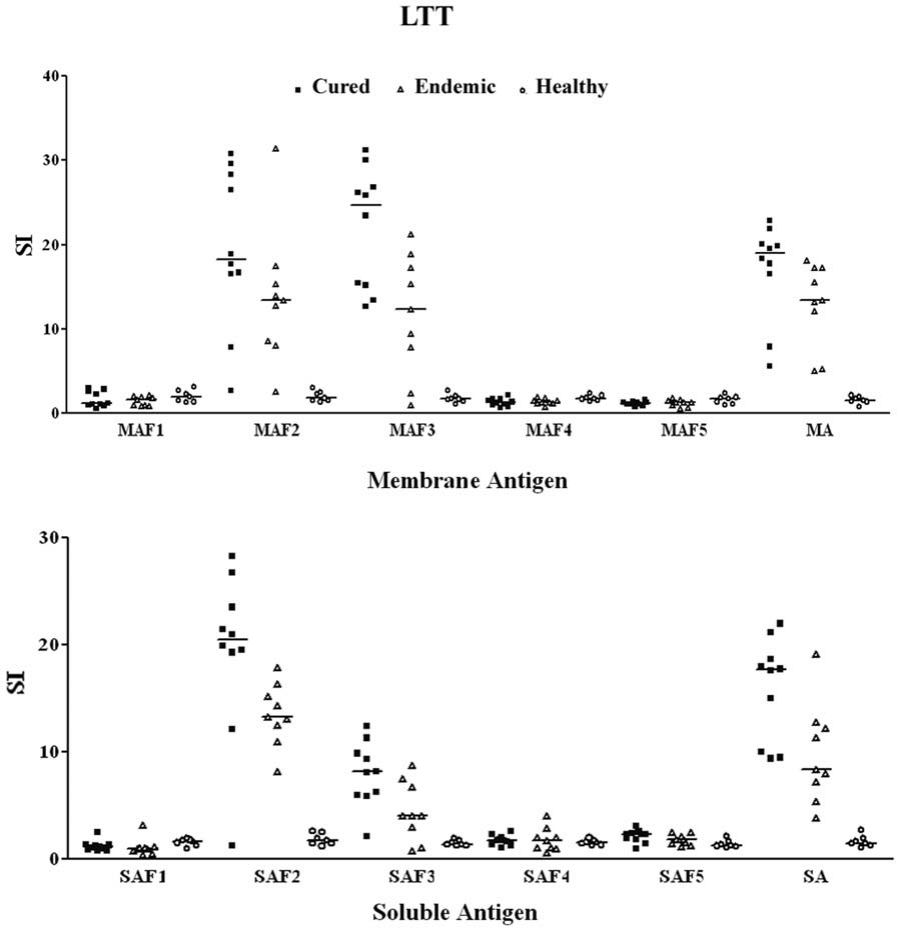
Lymphoproliferative response of PBMCs from Cured VL patients (▪), Endemic controls (▴) and healthy (o) individuals against membrane antigenic fractions/MA and soluble antigenic fractions/SA.

Since, in Indian kala-azar as well as in experimental models it was reported that impairment of cell-mediated immune response is reflected by marked T cell anergy specific to *Leishmania* antigens [40], [41], [42], we assessed the cellular responses in PBMCs of cured patients, endemic and non endemic healthy controls and *L. donovani* infected donors at a predetermined optimum concentration of 10 µg/ml. Individual donors were found to elicit different responses as compared to endemic control and cured patients. Endemic control and cured patients exhibited relatively higher SI index to the tune of 53.18±6.68 and 45.07±5.029 respectively against PHA as compared to unstimulated control (data not shown). The response to membrane sub-fractions MAF2 and MAF3 was nearly same and positive in 8 out of 10 cured *Leishmania* patients (mean SI value 19.57 with range of 2.7–30.80 for MAF2 and mean SI value 22.05 with range of 12.70–31.20 for MAF3) and in 8 out of 9 endemic controls (mean SI value 13.75 with range of 2.73–30.80 for MAF2; P<0.001 and mean SI value 11.76 with range of 1.03–21.30 for MAF3). Among the various soluble antigenic sub-fractions, SAF2 showed optimum proliferative response in cured (mean SI value 19.32 with range of 1.28–29.23; P<0.001) as well as endemic contacts (mean 13.54 with range of 8.2–17.9) (Fig. 4.) followed by SAF3 in soluble sub-fractions. Among the individual soluble sub-fractions SI values in cured/endemic for respective sub-fractions SAF1, SAF4, and SAF5 showed to be very poor response. Similar responses were observed with other three membrane sub-fractions.

There was no mitogenic as well as antigenic (all the individual sub-fractions as well as pooled one) proliferative response in *Leishmania* infected patients (data not shown).

### Estimation of cytokine (IFN-γ, IL-12p40 and IL-10) levels

The absence of a type 1 immune response to *Leishmania* antigen is documented in patients with VL along with activation of Th2-type cytokines resulting in progressive disease whereas Th1- type cytokine responses have been reported to play a critical role in protection against infection with *Leishmania* parasites (cured patients). Keeping this in view, the level of Th1 stimulatory i.e. IFN-γ & IL-12p40 as well as Th2 stimulatory IL-10 was estimated in PBMCs from cured patients as well as in endemic contacts against all the membrane and soluble subfractions. Optimum stimulation of IFN-γ and IL-12p40 responses was again noticed against subfraction MAF2, MAF3, SAF2 in 8 cured patients and 8 out of 9 endemic contacts followed by SAF3. The level of IFN-γ in cured patients stimulated by subfractions MAF2 and MAF3 was nearly same and positive in 8 out of 10 cured (593 pg/ml with range of 209–785 pg/ml for MAF2 and 510 pg/ml with range of 239–709 pg/ml for MAF3). Similarly, in endemic contacts IFN-γ level was 401 pg/ml with range of 236–645 pg/ml for MAF2 and 319 pg/ml with range of 189–574 pg/ml for MAF3. The level of IL-12p40 in cured patients stimulated by subfractions MAF2 and MAF3 was also nearly the same and positive in 8 out of 10 cured (530 pg/ml with range of 330–665 pg/ml for MAF2 and 628 pg/ml with range of 483–754 pg/ml for MAF3). Similarly, in endemic contacts IL-12p40 level were 449.7 pg/ml with range of 245–546 pg/ml for MAF2 and 506 pg/ml with range of 324–674 pg/ml for MAF3. The level of these cytokines was comparatively lower against the individual subfractions in the above stated study groups. Moreover, no detectable amount of IFN-γ and IL-12p40 level was observed with the lymphocytes of the six *L. donovani* infected patients (data not shown) and 7 healthy individuals with other antigenic sub-fractions.

**Figure 5:**
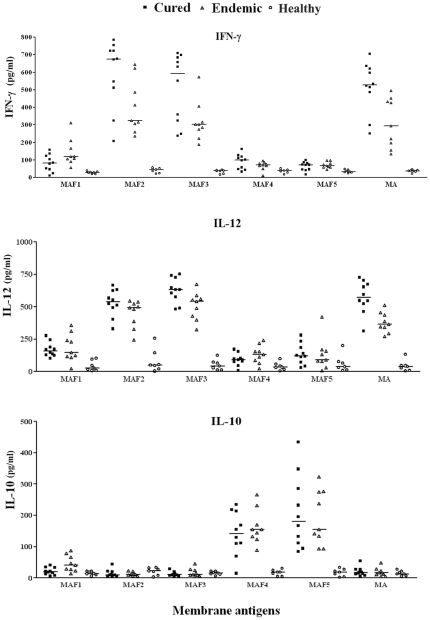
Th1/Th2 cytokine levels (IFN-γ, IL-12 p 40, IL-10) of PBMCs from cured VL patients (full course of treatment with Amphotericin B) (▪), endemic contacts (▴) and healthy (o) individuals against membrane antigenic fractions used at a concentration of 10 µg/ml (1 µg/well). The results are presented in the graph as for individual human sample along with horizontal bar representing the median.

The level of IFN-γ in cured patients stimulated by subfractions SAF2 was positive in 9 out of 10 cured patients followed by SAF3in which it was not very significant (534 pg/ml with range of 133–856 pg/ml for SAF2 and 180 pg/ml with range of 111–285 pg/ml for SAF3). Similarly, in endemic contacts IFN-γ level was 376 pg/ml with range of 121–621 pg/ml for SAF2. The level of IL-12p40 in cured patients stimulated by subfractions SAF2 and SAF3 was positive in all the cured patients(688.8 pg/ml with range of 507.2–879.7 pg/ml for SAF2 and 265 pg/ml with range of 163.6–349 pg/ml for SAF3). Similarly, in endemic contacts IL-12p40 level was 614 pg/ml with range of 430–868 pg/ml for SAF2. The level of these cytokines was comparatively lower against the individual subfractions in the above stated study groups. Moreover, no detectable amount of IFN-γ and IL-12p40 level was observed with the lymphocytes of the six *L. donovani* infected patients (data not shown) and 7 healthy individuals with other antigenic subfractions.(Fig. 5.)

**Figure 6:**
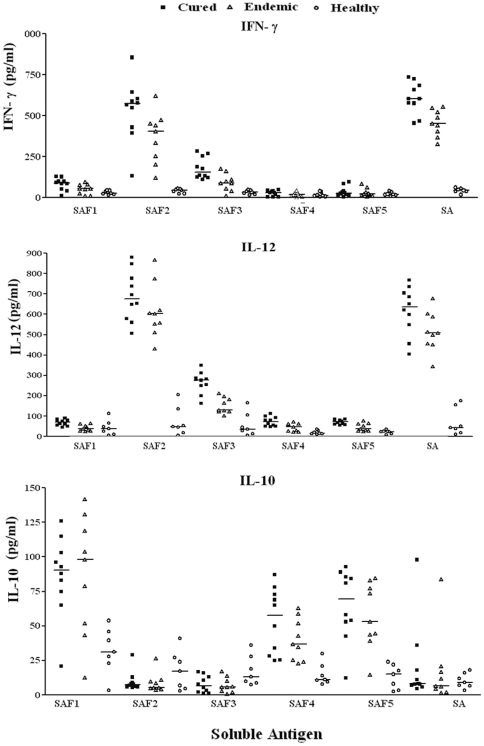
Th1/Th2 cytokine levels (IFN-γ, IL-12 p 40, IL-10) of PBMCs from cured VL patients (full course of treatment with Amphotericin B) (▪), endemic (▴) and healthy (o) individuals against soluble antigenic fractions used at a concentration of 10 µg/ml (1 µg/well). The results are presented in the graph as for individual human sample along with horizontal bar representing the median.

On the other hand low level of IL-10 cytokine against MAF2, MAF3, was found in cured patients (13.89 pg/ml with range of 4.02–44.03 pg/ml for MAF2 and 12.41 pg/ml with range of 5.32–28.93 pg/ml for MAF3) as well as in endemic contacts (12.77 pg/ml with range of 5.89–23.07 pg/ml for MAF2 and 16.93 pg/ml with range of 5.38–44.71 pg/ml for MAF3) was noticed (Fig. 6.). Level of IL-10 was also found very low in SAF2 and SAF3 subfractions in cured patients (9.76 pg/ml with range of 5.5–29 pg/ml for SAF2 and 7.80 pg/ml with range of 1.11–16.80 pg/ml for SAF3).In endemic controls also IL-10 was found in very low levels (8.92 pg/ml with range of 3.8–26.53 pg/ml for SAF2 and 7.24 pg/ml with range of 1.03–17.20 pg/ml for SAF3). But in all six infected patients IL-10 cytokine level was increased (data not shown) as compared to cured/endemic as well as healthy controls.

### MA and SA fractions and sub-fractions of amastigotes induced significant lymphoproliferative and NO responses in cured hamsters

In the previous experiment, the cellular responses of MAF2, MAF3 and SAF2 fractions assessed in human samples were observed to be the best among the membranous and soluble antigens. These were further validated/assessed for their lympho-proliferative responses in cured hamsters.

**Figure 7:**
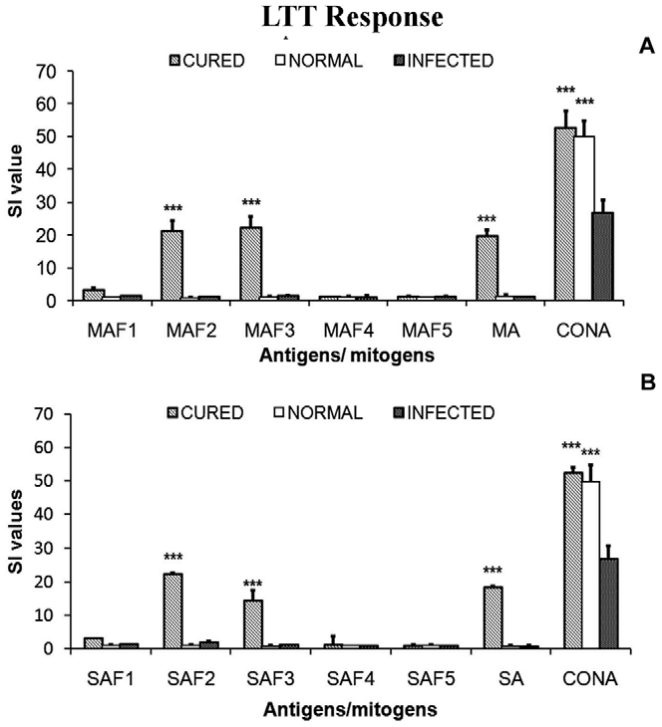
Lymphoproliferative response of mononuclear cells of lymph nodes from normal, *L. donovani* infected as well as cured (with Miltefosine at 40 mg/kgx5 days) hamsters in response against MA (A) and SA (B).

The normal control as well as cured *Leishmania* infected group had shown significantly higher proliferative responses against Con A. The SI values were 49.87±4.95 and 52.68±5.14 respectively (P<0.001). On the other hand, comparatively lower mitogenic (SI- 26.86±3.77) responses were observed in the cells from *L. donovani* infected group (Fig. 7). Among the fractions, the most significant lymphoproliferative responses in cured hamsters was observed against SAF2 (24.12±2.9, P<0.001) followed by SAF3 (16.67±1.53, P<0.001) and nearly equally potent response was observed in MAF2 (21.26±3.055) and MAF3 (22.21±3.33) fractions. Lymphoproliferative responses against other fractions were negligible. The LTT responses of the positive control SA and MA were also significantly higher to the tune of 18.46±1.46 (P<0.001) and 19.66±1.86 respectively which was almost parallel to its fractions.

**Figure 8:**
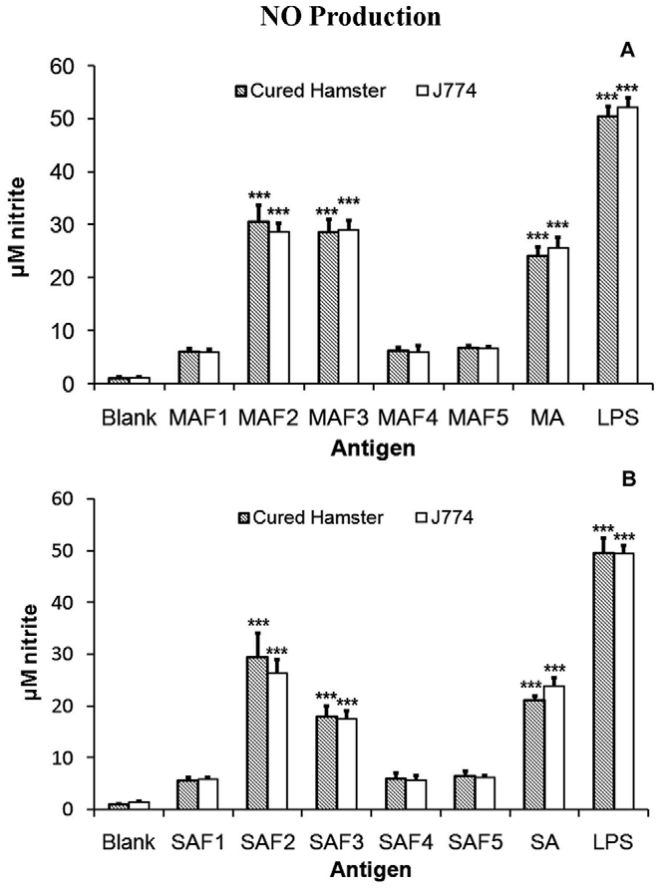
Nitric oxide production (µM) by peritoneal macrophages of hamsters (n = 9) and J774 cell lines in response to MA (A) and SA (B) at 10 µg/ml after 24 h stimulation. The absorbance of the reaction product was measured at 540 nm using Griess reagent.

Similar to LTT response, NO production in peritoneal macrophages of cured hamsters and J774A.1 macrophage cell lines was studied after 24 h of incubation in response to fractions MAF1-MAF5, SAF1-SAF5 and positive controls (MA, SA). For comparison, NO production in LPS (mitogen) stimulated and unstimulated cells served as positive and negative controls respectively. Among membrane fractions, MAF2 and MAF3 sub-fractions stimulated highest level of NO production in peritoneal macrophages of hamsters (30.58±3.10 µM; 28.63±2.31 µM) and J774A.1 (28.69±1.514 µM; 29.08±1.79 µM) cell line (Fig. 8.). However, there was almost negligible NO production against other sub-fractions in both the cell lines. In case of soluble sub-fractions, SAF2 stimulated highest production of NO in peritoneal macrophages of hamsters (29.40±4.69 µM) and J774A.1 (26.29±2.61 µM) cell line. It was followed by sub-fraction SAF3 with values being 17.86±2.13 and 17.45±1.66 in hamsters and J774.A1 cell lines respectively. However, there was almost negligible NO production against other sub-fractions in both the cell lines (data not shown) (Fig. 8).

## Discussion

Studies on the identification of novel vaccine and drug targets from amastigote stage of *Leishmania* spp are limited. This may be perhaps due to inadequate methods to isolate the intracellular form from host cell material. In this study, we used the classical method by Chang *et al* [21] to purify amastigotes and confirmed the purity of amastigotes microscopically using antibody raised against PPG [29]. Western blot analysis done with the antibodies specific for promastigote (LPG) and amastigote (A2) was to prove that the protein lysate used for the experiments in this study was of amastigotes. With these purified amastigotes, separated into membrane as well as soluble fractions, *in vitro* immunological studies were carried out which revealed that two fractions in the range of 97 to 68 kDa and 68 to 43 kDa have remarkable immunostimulatory potential.

Various approaches have been undertaken in order to identify the most relevant molecules as vaccine candidates against Leishmaniasis and one such approach consists of recognition of defined antigens by PBMCs or sera from patients or animals recovered from *Leishmania* infections [13], [32]. Few studies have been carried out to systematically evaluate biochemically-purified proteins and whole parasite lysate as immunoprotective agents for *Leishmania sp* [33], [34], [35], [36], [37], [38], most of which are restricted to promastigotes. It is therefore imperative to investigate the development of human T-cell responses to different *L.donovani* amastigote antigens which are of more importance being the pathogenic form of disease [39], [40]. One of the very few studies conducted using “axenic” amastigotes , identified three molecules (P-2/A-2, P-4 and P-8) expressing preferentially in the amastigote stage and further explored for immunization studies in BALB/c mice [41]. The finding that two of the three molecules provided significant protection against *Leishmania* infection was encouraging and strongly supported the view that, in general, antigens expressed by the amastigote stage are the potential source of vaccine candidates. This instigated us to identify immunostimulatory antigens in amastigotes isolated from heavily infected spleen of hamsters.

We fractionated amastigote antigen into membrane and soluble fractions as it is well-known that both contain potential immunogenic molecules. The membrane antigens are the first to interact with the vertebrate host's cells during the invasion strategy of the *Leishmania* parasite. Therefore, the molecules present on the cell surface are of paramount importance for designing the vaccines as these could check the multiplication of parasite by inactivating the parasitic invasion tools and also by triggering the cell mediated immune response [42], [43]. In addition, soluble proteins can also be effective as vaccine candidates since these can trigger immunological responses on recognition by T cells through antigen presentation by MHC class I and MHC class II grooves, regardless of their location in the parasite [44]. Further, since, these membrane as well as soluble proteins are a mixture of different antigenic proteins, it could stimulate different clones of B & T cells [45], [46]. Therefore, it is essential to identify specific TH1 stimulatory protective proteins from the Leishmania amastigote antigens through activity guided fractionation.

Using conventional screening approach, we have identified soluble fractions SAF2 followed by SAF3, which could stimulate the lymphocytes *in vitro* by inducing the release of very high amount of IFN-γ and IL-12p40, the molecules involved in cell-mediated protective immunity [47], [48], [49], [50], in cured patients/endemic contacts. Moreover, equally efficient immuno-protective response was obtained in membranous ones i.e. MAF2 and MAF3. However, rest of the antigenic sub-fractions did not induce proliferation or IFN-γ/IL12p40 production in healthy as well as in VL infected control subjects evaluated. On the other hand, the level of IL-10 against SAF2 and MAF2 as well as MAF3 followed by SAF3 was found suppressed in cured patients as well as in endemic contacts. These findings indicate a clear-cut and specific up-regulation of Th1 stimulatory responses with parallel down-regulation of Th2 stimulatory responses in cured patients. Noticeably in endemic controls the overall proliferative/cytokine responses of T-cells were not as high as observed in recovered ones. The presence of positive immune response in 60% of the endemic controls in this study supported the observation of Tripathi *et al* [51] that frequency of sub-clinical infections in an endemic area like Bihar is high which has also been reported in other areas of the world [52], [53]. The heterogeneity in the pattern of responses in recovered cases may have been attributed to the extent and duration of infection or the genetic restriction of the host anti-leishmanial T cells [54].

The immunogenicity data of these fractions and subfractions obtained with human PBMCs was further translated in hamster model. The reason behind taking hamster as experimental model was the fact that infection in golden hamsters [55] mimics several aspects of human disease, such as hepatosplenomegaly, pancytopenia, progressive cachexia, hypergammaglobulinemia, and suppression of T-cell proliferative response to parasite antigens [56]. Hence, it would be relevant to corroborate the observations on immunological responses of human lymphocytes with the animal model being used further for vaccination studies. In tune with the human data, almost all the cured animals too exhibited conspicuous stimulatory responses to the fractions SAF2, MAF2, MAF3 followed by SAF3 as evident by T cell proliferation. Recovery from *Leishmania* infection relies on induction of the Th1 response [57] with production of IFN-γ and IL-12 and enhanced expression of nitric oxide synthase [58]. Besides leishmanicidal activity, NO is also known to regulate immunological pathways including endogenous IL-12 secretion [59]. Therefore, in the absence of cytokine reagents for hamsters, nitric oxide assay was used to indirectly estimate the IFN-γ response, as NO is up-regulated by IFN-γ. The effect of the fractions on NO production was also validated in J774A-1 cell line.

Inference drawn from the overall immunogenic responses strongly suggests that the sub-fractions SAF2, MAF2 and MAF3 are the most immunopotents. Further, the cellular responses of SAF3 sub-fraction, though less as compared to other fractions, was remarkable. This suggest that these sub-fractions have some potent molecules that might have epitopes readily recognized by T-cells from both treated/cured as well as endemic individuals presumably sensitized with *L. donovani*. Besides, the cellular responses to potent fractions of both promastigotes [13], [32] and amastigotes of *L. donovani* were similar in endemic controls and in cured patients of VL as well as in hamsters, which would provide a strong basis for the hypothesis that the observations of vaccination studies of hamsters could be translated into humans.

To surmise, this finding leads to the first step of antigen selection in experimental models as well as in human being, in order to evaluate ability to induce protection against leishmanial infection. These *in vitro* findings need essential validation through vaccination studies in *in vivo* models to move ahead. Further, the results have also emphasized the necessity to dissect the potent fractions through proteomics to identify the specific proteins which are immunostimulatory.

## Materials and Methods

### Animal

Laboratory bred male golden hamsters (*Mesocricetus auratus*, 45–50 g) were employed for experimental work and maintained in plastic cages (38×27×13 cms) with standard rodent pellets diet (Lipton India Ltd.) and water *ad libitum*.

### Parasites

The *L. donovani* (strain 2001) were grown in RPMI-1640 medium (*in vitro*) supplemented with 10% heat-inactivated foetal bovine serum (Sigma, USA) at 25°C as described previously [13], [20]. *Leishmania* parasites have also been maintained in hamsters (*in vivo*) through serial passage, i.e., from amastigote to amastigote [13].

### Isolation of Amastigotes

Splenic amastigotes were isolated as per method described by Chang [21]. Briefly, the hamsters with well established infection (45–60 day old) were sacrificed, spleen removed aseptically in sterile Phosphate Buffer Saline (PBS) and cut into small pieces. The Splenic tissue was homogenized gently with the help of motor driven tissue homogenizer, consisting of glass mortar and Teflon pestle at 4°C. The suspension was centrifuged at 900×g for 10 min at 4°C, allowing the tissue debris to settle down. The supernatant was centrifuged at 3000×g for 20 min. at 4°C. The supernatant was discarded and the sediment containing amastigotes was re-suspended in RBC lysis solution to lyse the RBC. The suspension was centrifuged again at 3000×g at 4°C and pellet so obtained was dissolved in PBS-EDTA (2 mM), and then centrifuged at 3000×g at 4°C for 10 min. The amastigotes pellet so obtained was purified using percoll (Sigma, USA) density gradient centrifugation as described previously [21].

### Immunofluorescence using confocal microscopy

The purified amastigote suspension containing 10^5^ cells in 400 µl were adhered on poly-L-lysine coated cover slips and were fixed for 20 min in 4% paraformaldehyde in PBS (pH 7.2). Cells were washed 3 times with 200 µl of PBS-glycine (0.5%),further blocked with 100 µl of 1% BSA (Bovine Serum Albumin) for 30 minutes at room temperature and incubated with a 100 µl of rabbit polyclonal antiserum raised against dephosphorylated proteophosphoglycan (PPG) (1∶200 diluted) for 30 minutes at room temperature . The cells then were again washed 6 times with 100 µl of 1% BSA and incubated with 100 µl of fluorescein isothiocyanate (FITC)-conjugated goat anti-rabbit IgG antibodies (Bangalore Genei, India)(1∶500 dilution) for 1 hour at 4°C in dark humid chamber.

### Western Blot analysis of pure amastigotes

The whole cell lysate (WCL) of splenic amastigotes as well as promastigotes of clinical isolate of *L. donovani* was prepared by boiling 10^7^ cells in 100 µl of 2× sample buffer (100 mM Tris-HCl, 200 mM DTT, 4% SDS, 0.2% bromophenol blue, 20% glycerol) for 10 min in water bath and chilled on ice. Then the samples were centrifuged at 8000×g for 10 minutes and 25 µl (300 µg/lane) of supernatant was separated on two sets of SDS-PAGE containing 6% stacking and 10% separating gels. One set of gel was stained with Coomassie blue and the other was used for transferring proteins to nitrocellulose membrane using Hoefer Semi-dry transfer assembly at 0.8 mA/cm2 of NC membrane [22], [23]. Successful transfer of the proteins was verified by the transfer of the pre-stained molecular weight standards (Fermentas, USA). Following overnight blocking of non-specific binding sites in 5% skimmed milk, NC membrane was probed with different antibodies viz. A2 (Medimabs, Montreal, Canada), LPG (GenWay Biotech, San Diego, CA) followed by incubation with horseradish peroxidase (HRP) conjugated goat anti-rabbit antibodies (Bangalore Genei, India) (1/5000 dilution). Grp78 protein (from Dr Emanuela Handman, Australia) was used as loading control. After washing with PBS-T (0.05% Tween-20), the protein bands detection was performed in dark, using the Amersham ECL kit of enhanced chemiluminescence (Amersham Biosciences, Singapore). Briefly, the NC membrane was incubated for 5 minutes with detection solutions (A and B at 40∶1 ratio) and then the membrane was wrapped up with SaranWrap and was placed in an x-ray film cassette. A sheet of autoradiography film (Amersham Biosciences, Singapore) was then placed on the top of the membrane and cassette was closed and the membrane was exposed for 10 minutes. Finally, the membrane was developed by developer and was fixed by the fixer.

### Fractionation of *L. donovani* amastigote proteins

#### Soluble protein (SA)

Soluble *L. donovani* amastigote antigen (SA) was prepared as per method described by Scott et al., [24] and modified by Choudhry et al., [25]. Briefly, isolated amastigotes were suspended in PBS and sonicated (Soniprep-150) for two periods of 1.5 min each in ice, (separated by an interval of 3 min) at medium amplitude. The sonicated sample was rapidly frozen and thawed 4 times using liquid nitrogen and left at 4°C for one hour for complete extraction of soluble antigen. The suspension was centrifuged at 4,000 g for 20 min at 4°C. The supernatant so obtained was finally ultra centrifuged at 40,000 g for 30 min. After assessing protein contents (Bradford method), the antigen was distributed in small aliquots and stored at −70°C.

#### Membrane protein (MA)

Membrane antigen (MA) was prepared using the method of Molloy *et al.* [26]. Briefly, amastigotes were disintegrated using freeze-thawing in liquid nitrogen and then lysed by ultrasonication in TNE buffer (50 mM Tris-HCl, pH 7.4, 150 mM NaCl, 5 mM EDTA) with Protease inhibitor cocktail (Sigma, USA). The lysate was then incubated with 0.1 M sodium carbonate for 1 h at 4°C. Finally, MA was collected by ultracentrifugation in a Beckman ultracentrifuge (CA, USA) at 120000×g for 1 h at 4°C. Such a pre-fractionation of the proteins reduces the complexity of 2-D maps by removing most non-membrane proteins [27].

### Sub-fractionation of SA and MA proteins in to five sub-fractions (MAF1-MAF5, SAF1-SAF5) by continuous-elution gel electrophoresis

SA as well as MA were prepared and resolved by 12% SDS-PAGE as described earlier. Resolved fractions of interest were identified with the help of pre-stained molecular weight markers run simultaneously and cut using sharp and clean scalpel. The bands were designated as F1 to F5. Proteins from gel strips were electro eluted (Electroeluter, Millipore, India), concentrated (Centricon of 3 10 and 30 kDa cut off; Millipore, India), and content estimated (Bradford method) . The molecular weight of the proteins in fractions was confirmed in 1D SDS-PAGE as above and then stored in aliquots at −20°C till use.

### Donors and isolation of mononuclear cells

A brief account of clinical samples has been given in Table 1. The study groups of clinical blood samples taken were as follows:

- **Ten** treated/cured patients from Kala-azar Medical Research Centre, Muzzafarpur (hyper-endemic area of Bihar) who had recovered from VL. Seven were males and three females, their age ranging from 2 to 32 years. Diagnosis was established in all cases by demonstration of parasites in splenic aspirates. All patients had received full course of Amphotericin B (Fungizone). Samples were collected two months to one year after completion of treatment. Their splenic aspirates were negative for parasites at the time of study
- **Nine** endemic household contacts, i.e. 5 males and 4 females, their age ranging from 20–58 years, were taken from Kala-azar Research centre. They neither showed any clinical symptoms nor received any anti leishmanial treatment.
- **Six** infected patients viz. 2 males and 4 females belonging to *Leishmania* endemic area were taken from the above stated centre; their age ranging from 5 to 20 years, showing clinical symptoms and presence of parasites in their splenic aspirates.
- **Seven** normal healthy donors were taken from non endemic areas. There were 6 males and one female, their age ranging from 25 to 30 years. They served as negative control as they never had any exposure to *Leishmania* infection and also have never travelled to *Leishmania* endemic area.

**Table 1:** Size and type of clinical samples taken for the study.

	Healthy controls	Infectedcontrols	Endemic controls	Recovered/cured patients
Number	7	6	9	10
Age (Yrs)	25–30	5–20	20–58	2–32
Male/Female	6/1	2/4	5/4	7/3
Treatment	Nil	Nil	Nil	Amphotericin

Approximately 10 ml of heparinized venous blood was collected from all the study subjects. Blood was mixed with equal amount of PBS (1.5 M, pH 7.4) and then 10 ml of diluted blood was layered on 5 ml (i.e. 2: 1 ratio) Ficoll Hypaque (Histopaque 1077- Sigma, USA). PBMCs were then isolated by centrifugation at 1000×g at room temperature for 30 min. The isolated PBMCs were washed three times with incomplete RPMI medium at 1000×g for 15 min, 900×g for 12 min and 900×g for 10 min. A final suspension of 1×10^6^ cells/ml was made with complete RPMI medium [HEPES-buffered RPMI-1640 supplemented with streptomycin (100 µg/ml), Penicillin (100 U/ml), L-glutamine (2 mM), β-mercaptoethanol (5×10^−5^ M) and 10% heat inactivated fetal bovine serum] after determining cell viability by trypan blue staining method (Tripathi et al., 2006).

### Treatment of *L. donovani* infected hamsters with antileishmanial drug and isolation of mononuclear cells

Approximately 30 hamsters, infected with 10^7^ amastigotes intracardially, were assessed for parasitic burden one month later by splenic biopsy through a small incision in the upper left quarter of the abdomen and a small piece of splenic tissue was cut and dab smears were made on slides. The incised portion was stitched with nylon suturing thread. Following biopsy, an adequate amount of antibiotic powder (Neosporin, Burroughs Wellcome Ltd., India) was applied on the stitched portion and finally sealed with tincture of benzoin. In addition, neosporin sulphate (100 mg/kg of body weight) was also given orally the day before and the day after the biopsy for healing. The smears were fixed in methanol and stained with Giemsa stain and the number of amastigotes/1000 cell nuclei was counted. The animals harbouring >20–25 amastigotes/100 macrophage cell nuclei were then treated with Miltefosine-an antileishmanial drug (Zentaris, Germany) at 40 mg/kg bodyweight daily for 5 days. The animals were reassessed for complete cure by splenic biopsy performed on day 30 post treatment. Lymph nodes (inguinal and mesenteric) from individual infected, treated/cured as well as normal hamsters were removed aseptically and placed in a petridish containing incomplete RPMI (iRPMI) medium. After 3–4 washing in iRPMI medium, lymph nodes were teased with sterile needles and then crushed with sterile pestle in petridishes. The suspensions so obtained were transferred into the sterile centrifuge tubes and centrifuged at 1000×g for 5 minutes to settle down the debris if any. Supernatants were re-centrifuged in another tube at 1500×g for 10 min. to spin down the lymphocytes. The cells were washed thrice with iRPMI and a final suspension of 1×10^6^cells/ml was made in cRPMI medium.

### Lymphoproliferative responses in cured/endemic patients and cured hamsters against five fractions

Lymphocyte proliferation assay was performed to evaluate cell-mediated immune response of cured/endemic patients and cured hamsters to leishmanial antigens. Suspension of PBMCs (1×10^6^/ml) of both patients and hamsters was cultured in 96 well flat bottom tissue culture plates (Nunc, Denmark). About 100 µl of mitogens - PHA (10 µg/ml Sigma, USA), Con A (10 µg/ml, Sigma, USA) as well as antigens i.e. subfractions SAF1 to SAF5 and MAF1 to MAF5 (10 µg/ml each), predetermined concentration, were added to the wells in triplicate. For comparison, SA and MA (10 µg/ml each) were employed as positive controls. Wells without stimulants served as negative control. Cultures were incubated at 37°C in a CO_2_ incubator with 5% CO_2_ for 3 days in the case of the mitogens, and for 5 days in the case of the antigens. Eighteen hours prior to termination of culture, 0.5 µCi of [^3^H]-Thymidine (BARC, India) was added to each well .They were then harvested on glass fibre mats (Whatman) and counted in a liquid scintillation counter. Results were expressed as stimulation index (SI) which was calculated as mean counts per minute (cpm) of stimulated culture/mean cpm of unstimulated control. SI values of unstimulated control group were compared with the values of stimulated (with antigen) groups and SI of more than 2.5 was considered as positive response.
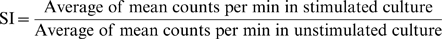



### Estimation of level of cytokines (IFN-γ/IL-12p40/IL-10) in lymphocytes of cured/endemic patients against five fractions

As mentioned above, culture of PBMCs from human patients was set up in 96 well culture plates and 1×10^6^ cells/ml were dispensed and antigenic fractions were added at a concentration of 10 µg/ml in triplicate wells with a final concentration of 1 µg/well. IFN-γ IL-12p40 and IL-10 levels in the 24 hrs antigen stimulated supernatants were determined by enzyme-linked immunosorbent assay kit (OptEIA set, Pharmingen). The results were expressed as picograms of cytokine/ml, based on the standard curve of the standards of respective cytokine provided in the kit. The lower detection limits for various cytokines were as follows: 5.1 pg/ml for IFN-γ, 30.8 pg/ml for IL-12p40 and 6.9 pg/ml for IL-10.

### Assessment of NO production in macrophages against five sub-fractions

The presence of NO was assessed using Griess reagent in the culture supernatants of naïve hamster peritoneal macrophages as well as macrophage cell line J774.1 after the exposure with supernatant of stimulated lymphocyte's cultures and LPS (10 µg/ml Sigma, USA) was used as mitogen (Sigma) [28]. Isolated peritoneal macrophages were suspended in culture medium and plated at 10^6^ cells/well and exposed to the supernatants of above described 5-day-old antigen stimulated lymphocyte's cultures from all the study groups. The supernatants (100 µl) collected from macrophage cultures 24 h after incubation was mixed with an equal volume of Griess reagent (Sigma, USA) and left for 10 min at room temperature. The absorbance of the reaction was measured at 540 nm in an ELISA reader.

### Ethics Statement

The study and the protocol was approved by the Ethics committee of the Kala-azar Medical Research Centre, Muzzafarpur and CDRI and all patients provided a written consent before enrolment to this study. Experiments on the animals (hamsters) were performed following the approval of the protocol and the guidelines of Institutional Animal Ethics Committee (IAEC) of the CDRI. The approval reference number is 126/08/Para/IAEC dated 27.08.2008

### Statistical analysis

The results (pooled data of three experiments) were analyzed by one-way ANOVA followed by Tukey's post test where appropriate. All of the analyses were done using GraphPad Prism (version 3.03) software. The upper level of significance was chosen as P<0.001.
